# Infestation of chigger mites on Chinese mole shrew, *Anourosorex squamipes*, in Southwest China and ecological analysis[Fn FN1]

**DOI:** 10.1051/parasite/2022038

**Published:** 2022-07-28

**Authors:** Bei Li, Xian-Guo Guo, Cheng-Fu Zhao, Zhi-Wei Zhang, Rong Fan, Pei-Ying Peng, Wen-Yu Song, Tian-Guang Ren, Lei Zhang, Ti-Jun Qian

**Affiliations:** 1 Institute of Pathogens and Vectors, Yunnan Provincial Key Laboratory for Zoonosis Control and Prevention, Dali University Dali Yunnan 671000 PR China; 2 Institute of Microbiology, Qujing Medical College Qujing Yunnan 655000 PR China; 3 Nursing College of Dali University Dali Yunnan 671000 PR China

**Keywords:** Acari, Mite, Trombiculidae, Ectoparasite, Insectivore, Soricidae

## Abstract

The Chinese mole shrew, *Anourosorex squamipes* Milne-Edwards, 1872, is a common species of insectivorous mammal in Southwest China. Based on field investigations between 2001 and 2019, the present study reports the infestation of chiggers (larvae of chigger mites) on the shrew in Southwest China and certain ecology parameters for the first time. A total of 3169 chiggers were collected from 1694 *A. squamipes* and they were identified into 72 species and 10 genera in the family Trombiculidae. The overall infestation prevalence (*P*_*m*_), mean abundance (*MA*) and mean intensity (*MI*) of *A. squamipes* with chiggers reached 11.1%, 1.87 and 16.86, respectively. The species diversity, species composition and infestation of chiggers on *A. squamipes* fluctuated in different environments (latitudes, altitudes, habitats and landscapes) and on different sexes and ages of the shrew hosts with high heterogeneity and low species similarity. In the established linear regression equation (M* = 0.173 + 1.054 *M*) for dominant mite *Leptotrombidium densipunctatum*, both the *α* and *β* values (*α* = 0.173, *β* = 1.054) exceeded the boundary values (*F* = 4.67, *p* < 0.05), and therefore the spatial distribution pattern of this mite was determined as an aggregated distribution among different individuals of shrew hosts. The species abundance distribution of the chigger community on *A. squamipes* conformed to the lognormal distribution, and its curve showed a gradually descending tendency from the rare mite species to the dominant mite species. The curve tendency of species-sample relationship implies that more species of chiggers would be found if the host samples infinitely keep increasing.

## Abstract (fr)

La musaraigne *Anourosorex squamipes* Milne-Edwards, 1872 est une espèce commune de mammifère insectivore dans le sud-ouest de la Chine. Sur la base d’enquêtes de terrain entre 2001 et 2019, la présente étude signale pour la première fois l’infestation et certains paramètres écologiques des acariens larvaires sur cette musaraigne dans le sud-ouest de la Chine. Un total de 3169 acariens ont été collectés sur 1694 *A. squamipes* et ils ont été identifiés en 72 espèces et 10 genres de la famille des Trombiculidae. La prévalence globale de l’infestation (*P*_*m*_), l’abondance moyenne (*MA*) et l’intensité moyenne (*MI*) des acariens sur *A. squamipes* ont atteint respectivement 11,1 %, 1,87 et 16,86. La diversité des espèces, la composition des espèces et l’infestation des acariens sur *A. squamipes* ont fluctué selon différents environnements (latitudes, altitudes, habitats et paysages) et selon le sexe et l’âge des hôtes avec une forte hétérogénéité et une faible similitude entre espèces. Dans l’équation de régression linéaire établie (M* = 0,173 + 1054 *M*) pour l’acarien dominant *Leptotrombidium densipunctatum*, les valeurs *α* et *β* (*α* = 0,173, *β* = 1,054) dépassaient les valeurs limites (*F* = 4,67, *p* < 0,05), et par conséquent, le modèle de distribution spatiale a été déterminé comme une distribution agrégée entre différents individus d’hôtes. La distribution de l’abondance des espèces de la communauté d’acariens sur *A. squamipes* se conformait à la distribution log-normale et la courbe a montré une tendance progressivement descendante des espèces d’acariens rares à l’espèce d’acarien dominante. La tendance de la courbe de la relation espèce-échantillon implique que davantage d’espèces d’acariens seraient trouvées si l’échantillonnage des hôtes continuaient à augmenter à l’infini.

## Introduction

Chigger mites (trombiculid mites) are a large group of tiny arthropods and are distributed worldwide [34, 37, 48]. The taxonomic status of chigger mites is controversial. In some of the literature, all the chigger mites have been grouped into one family, the family Trombiculidae, in the suborder Prostigmata under the order Trombidiformes of the superorder Actinotrichida (or Acariformes) [25, 34, 50]. In other articles, however, the chigger mites have been placed in two families (Trombiculidae and Leeuwenhoekiidae) or three families (Trombiculidae, Leeuwenhoekiidae and Walchiidae) under the order Trombidiformes of the superorder Acariformes [4, 37, 53, 58]. To date, more than 3000 species of chigger mites have been recorded in the world and more than 500 species documented in China [5, 37, 63, 68]. The life cycle of chigger mite is complex with seven basic stages: the egg, prelarva (deutovum), larva, protonymph (nymphochrysalis), deutonymph (nymph), tritonymph (imagochrysalis) and adult (male and female) [25, 38, 48]. The larval stage of chigger mite is often known as “chigger” and therefore it is called “chigger” in the present paper instead of “chigger mite”. The chigger is the only ectoparasitic stage (ectoparasite) on the body surface of some other animals (hosts) and the exclusive transmitting vector of scrub typhus (tsutsugamushi disease) caused by *Orientia tsutsugamushi* [2, 25, 48]. Besides transmitting scrub typhus, some chigger species can be potential vectors of Hemorrhagic Fever With Renal Syndrome (HFRS) caused by Hantavirus [12, 42, 67], and some chiggers may be associated with the transmission of *Borrelia burgdorferi* and *Rickettsia connori* in Europe [2, 8, 27]. As a common group of ectoparasites, chiggers usually have a wide range of hosts such as reptiles, birds and mammals, including humans. Small mammals, especially rodents and shrews, are the most common hosts of chiggers [25, 35]. Chiggers usually climb and adhere to a certain height of the vegetation surface or gather at a high degree of depression hidden on the ground, waiting for the appropriate time to attach to the host animals [35, 63].

The Chinese mole shrew, *Anourosorex squamipes* (Milne-Edwards, 1872), is a common species of insectivore (Eulipotyphla: Soricidae), and it is also called mole shrew or Sichuan burrowing shrew, which is widely distributed in Southwestern China, Northern Vietnam, Northern Thailand and Bhutan [57, 60]. As a species of insectivorous mammal, *A. squamipes* often preys on soil arthropods, worms, young rodents and aquatic animals, and it plays an important role in maintaining the ecological balance in the food chain [17, 36]. At the same time, however, *A. squamipes* also eats various plants and crops (including their seeds and stems) and damages them as an agricultural and forestry pest, and it has two sides of function in the agroecosystem because of its complex feeding habits [22, 36, 73]. Moreover, *A. squamipes* is also an important reservoir host of some zoonoses (zoonotic diseases) such as leptospirosis and hantavirus lung syndrome (HPS), and it is of medical importance [9, 11, 29]. In Southwest China, *A. squamipes* is a common species of small mammal with a large population and it is often an agricultural and medical pest in the region [17, 18, 22]. Although previous studies reported the mitochondrial genome determination, biological characteristics and population dynamics of *A. squamipes* shrew [17, 18, 22, 26, 66, 73], few articles have involved the infestation of the shrew with ectoparasites (including chigger mites) and the related ecology of the mites. Between 2001 and 2019, our research group carried out a long-term field investigation and accumulated abundant original data on chiggers in Southwest China which includes five provincial regions (covering 24.5% of China’s land area), Sichuan, Yunnan, Guizhou, Tibet (Xizang Autonomous Region) and Chongqing [71]. To fully take advantage of the investigation data, the present study comprehensively analyzed the infestation and the related ecology of chiggers on *A. squamipes* in Southwest China based on the basic strategy of “data mining”. The present paper is the first to study chiggers on *A. squamipes* shrew across the five provincial regions of Southwest China, which is an attempt to enrich the knowledge about the shrew and its ectoparasitic chiggers, and provide some scientific information for the surveillance of chiggers and other related studies.

## Materials and methods

### Field investigation and collection of shrews and chigger mites

**Figure 1:**
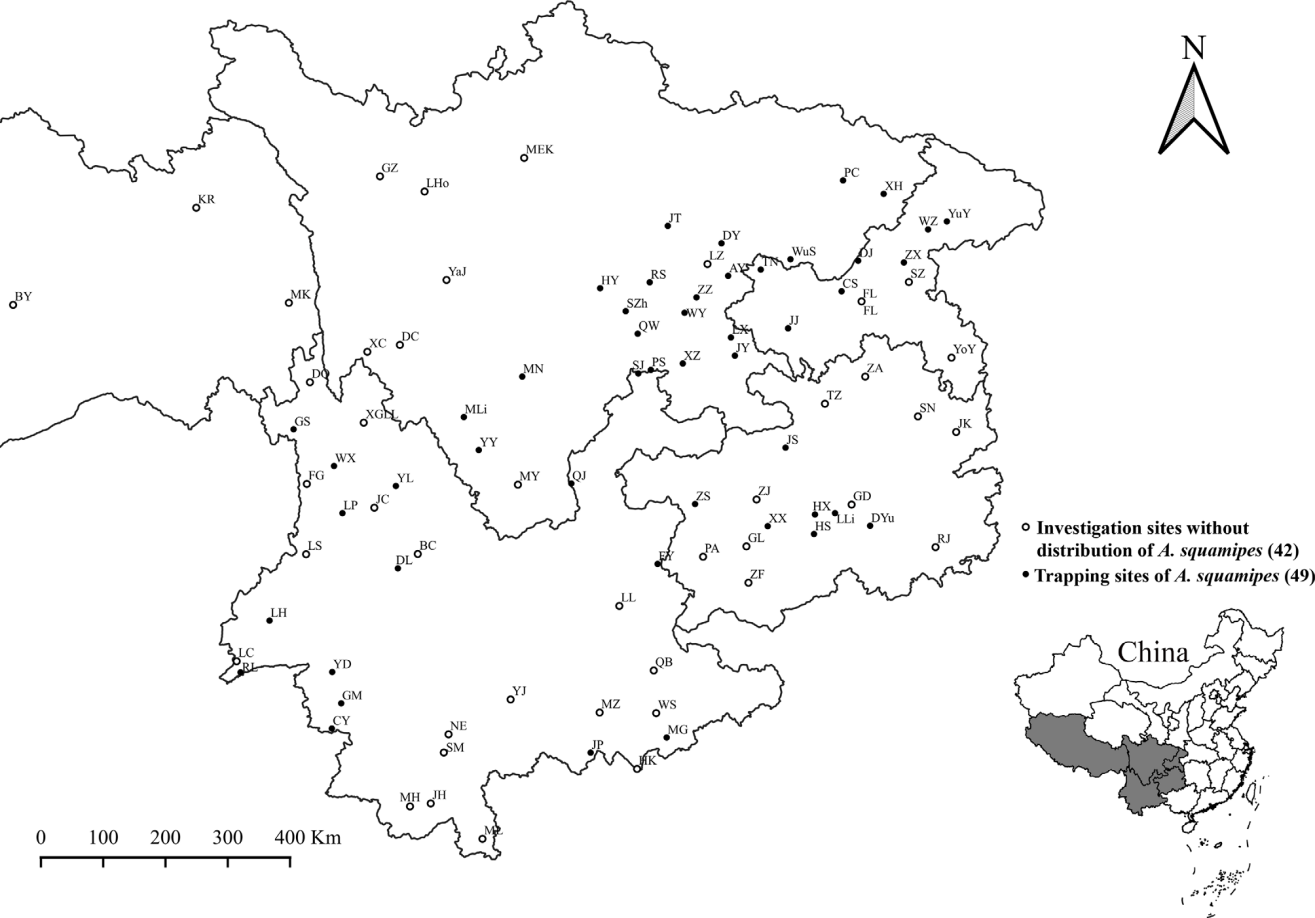
Investigation sites (*n* = 91) in Southwest China between 2001 and 2019.

**Figure 2:**
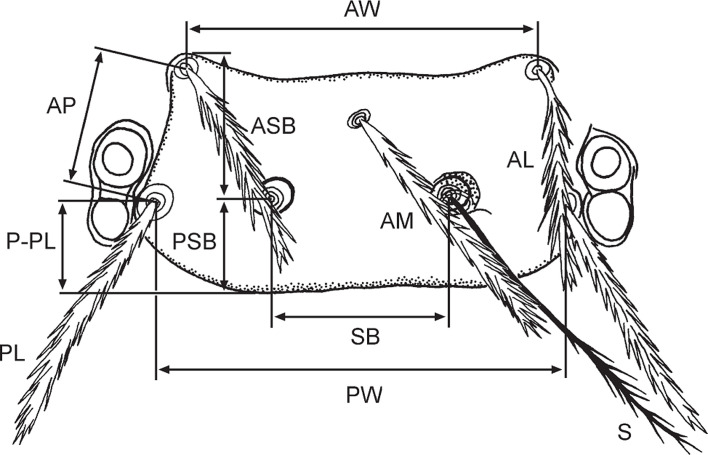
Scutum of chiggers and measurements (abbreviations and corresponding morphology are the same as in Table 2, cited from Stekolnikov, 2013 [49]).

The field investigation was carried out at 91 investigation sites (counties) in Southwest China (see Table 1 and Fig. 1 in “Results”). Shrews and other small mammals were captured with mousetraps (18 × 12 × 9 cm, Guixi Mousetrap Apparatus Factory, Guixi, Jiangxi, China). At the investigation sites, mousetraps with baits were randomly placed in different habitats in the evening and then checked the next morning. The habitats covered residential areas (houses, stables, barns and nearby surroundings), farmlands, bushes and woodlands. The trapped shrews and other small mammals were separately placed in a white cloth bag and then transported to the field laboratory where ectoparasitic chiggers were collected. The collected chiggers were preserved in a vial containing 70% ethanol [11, 38, 68]. After the collection of chiggers, each animal host was identified into species according to its appearance (body size, shape and hair color), body measurements (body weight, body length, tail length, ear height and hind foot length) and other morphological characteristics [56, 60, 61]. In the laboratory, the collected chiggers were mounted onto glass slides with Hoyer’s solution. After dehydration, drying and transparency, the mounted specimens of chiggers were identified into species under Leica DM 3000 LED microscopes [11, 13, 21, 25, 49]. According to the available taxonomic literature (books and papers) and taxonomic keys, the identification for each chigger specimen was done under a high power lens (10 × 40) and oil immersion lens (10 × 100) of the microscope, based on the careful observation and measurement of the related taxonomic structures of chiggers (see Table 2 and Fig. 2 in “Results”) [25, 49, 50, 53]. Following identification of animal hosts and chiggers, all Chinese mole shrews (*A. squamipes*), together with chiggers on the body surface of shrews, were chosen as the target of the present study. The use of animals (including animal euthanasia) for research was officially approved by the Animal Ethics Committee of Dali University, under permission number DLDXLL2020-1104. Representative specimens of animal hosts (shrews) and chiggers were deposited in the specimen repository of the Institute of Pathogens and Vectors, Dali University, Dali, Yunnan, China.

**Table 1:** A total of 91 investigation sites (counties) in Southwest China (2001–2019).

No.	Codes	Investigation sites
1	AY	Anyue*
2	BC	Binchuan
3	BY	Bayi (Linzhi city)
4	CS	Changshou*
5	CY	Cangyuan*
6	DC	Daocheng
7	DJ	Dianjiang*
8	DL	Dali*
9	DQ	Deqin
10	DY	Daying*
11	DYu	Duyun*
12	FC	Fucheng (Mianyang city)*
13	FG	Fugong
14	FL	Fuling
15	FY	Fuyuan*
16	GD	Guiding
17	GL	Guanling
18	GM	Gengma*
19	GS	Gongshan*
20	GZ	Ganzi
21	HK	Hekou
22	HS	Huishui*
23	HX	Huaxi (Guiyang city)*
24	HY	Hongya*
25	JC	Jianchuan
26	JH	Jinghong
27	JJ	Jiangjin*
28	JK	Jiangkou
29	JP	Jinping*
30	JS	Jinsha*
31	JT	Jintang*
32	JY	Jiangyang (Luzhou city)*
33	KR	Karuo (Changdu city)
34	LC	Longchuan
35	LH	Lianghe*
36	LHo	Luhuo
37	LL	Luliang
38	LLi	Longli*
39	LP	Lanping*
40	LS	Lushui
41	LX	Luxian*
42	LZ	Lezhi
43	MEK	Maerkang
44	MG	Maguan*
45	MH	Menghai
46	MK	Mangkang
47	ML	Mengla
48	MLi	Muli*
49	MN	Mianning*
50	MY	Miyi
51	MZ	Mengzi
52	NE	Ninger
53	PA	Puan
54	PC	Pingchang*
55	PS	Pingshan*
56	QB	Qiubei
57	QJ	Qiaojia*
58	QW	Qianwei*
59	RJ	Rongjiang
60	RL	Ruili*
61	RS	Renshou*
62	SJ	Suijiang*
63	SM	Simao
64	SN	Sinan
65	SZ	Shizhu
66	SZh	Shizhong (Leshan city)*
67	TN	Tongnan*
68	TZ	Tongzhi
69	WS	Wenshan
70	WuS	Wusheng*
71	WX	Weixi*
72	WY	Weiyuan*
73	WZ	Wanzhou*
74	XC	Xiangcheng
75	XGLL	Xianggelila
76	XH	Xuanhan*
77	XX	Xixiu (Anshun city)*
78	XZ	Xuzhou (Yibin city)*
79	YaJ	Yajiang
80	YD	Yongde*
81	YJ	Yuanjiang
82	YL	Yulong*
83	YoY	Youyang
84	YuY	Yunyang*
85	YY	Yanyuan*
86	ZA	Zhengan
87	ZF	Zhenfeng
88	ZJ	Zhijin
89	ZS	Zhongshan (Liupanshui city)*
90	ZX	Zhongxian*
91	ZZ	Zizhong*

Note: The investigated sites (counties) marked with “*” were the capture sites for Chinese mole shrews (*Anourosorex squamipes*).

**Table 2:** The related taxonomic morphology of chiggers, the larvae of chigger mites (cited from Stekolnikov, 2013 [49]).

Abb.	Corresponding morphology
AL	Length of anterolateral scutal setae
AM	Length of anteromedian scutal seta
AP	Distance from AL to PL on one side
AW	Distance between anterolateral scutal setae
*D*_max_	Length of the longest dorsal idiosomal seta
*D*_min_	Length of the shortest dorsal idiosomal seta
DS	Number of dorsal idiosomal setae (including humeral)
fCx	Coxal setation
fT	Formula of palpotarsus
H	Length of humeral setae
Ip	Sum of leg lengths (pa + pm + pp)
NDV	Number of idiosomal setae (DS + VS)
Oc	Eyes on ocular plates
ASB	Distance from the level of sensillary bases to extreme anterior margin of scutum
fsp	Leg segmentation formula, the numbers of apparent segments of each pair of legs
fD	Dorsal setal formula including number of humeral setae (H) and arrangement of dorsal idiosomal setae by rows
fSt	Sternal setal formula, the numbers of anterior (between coxae I) and posterior (between coxae III) sternal setae
fp	Palpal setation formula (N = nude, B = branched) including conditions of palpal femoral seta, palpal genual seta, and three palpal, tibial setae (dorsal, lateral, and ventral)
pa	Length of leg I (including coxa)
PL	Length of posterolateral scutal setae
pm	Length of leg II (including coxa)
pp	Length of leg III (including coxa)
PW	Distance between posterolateral scutal setae
S	Length of sensilla
SB	Distance between sensillary bases
SD	Length of scutum (ASB + PSB)
TaIIIL	Length of leg tarsus III
TaIIIW	Width of leg tarsus III
*V*_max_	Length of the longest ventral idiosomal seta
*V*_min_	Length of the shortest ventral idiosomal seta
PLs	The horizontal line of the base of posterolateral scutal setae
VS	Number of ventral idiosomal setae (excluding coxal and sternal)
PSB	Distance from the level of sensillary bases to extreme posterior margin of scutum
SIF	Synthetic identification formula which is generally accepted in the taxonomy of chigger mites
P-PL	Distance from the level of posterolateral scutal setae to extreme posterior margin of scutum

### Statistics of chigger infestation on *A. squamipes*

The constituent ratio (*C*_*r*_), prevalence (*P*_*m*_), mean abundance (*MA*), and mean intensity (*MI*) were used to calculate the infestation of *A. squamipes* with chiggers. Differences in infestation were compared based on different sexes and ages of hosts (*A. squamipes*), and on different latitudes, altitudes, habitats and geographical landscapes [13, 30, 40, 45, 51, 62, 68]. The formulae of *C*_*r*_, *P*_*m*_, *MA* and *MI* were as follows:

$$C_{r}=\frac{N_{m}}{M}\times 100\%;$$

$$P_{m}=\frac{H_{m}}{H}\times 100\%;$$

$$\mathrm{MA}=\frac{N_{m}}{H};$$

$$\mathrm{MI}=\frac{N_{m}}{H_{m}}.$$

In the above formulae, *N*_*m*_ = the individuals of a certain chigger species, *M* = the total individuals of all the chigger species, *H*_*m*_ = the individuals of *A. squamipes* shrews infested with chiggers, and *H* = the individuals of all the shrews examined.

### Statistics of chigger community on *A. squamipes*

In the present study, all the chiggers on *A. squamipes* are defined as a chigger community. Species richness (*S*), Shannon–Wiener diversity index (*H*′), Pielou evenness index (*E*), and Simpson dominance index (*D*) were used in the calculation of the community. Jaccard’s similarity index (*J*) was used to compare the species similarity between two different community units [14, 31, 47, 54, 62, 72].

$$S=\sum S_{m};$$

$$H^{\prime }=-\sum_{m=1}^{S}\left(\frac{N_{m}}{M}\right)\ln\left(\frac{N_{m}}{M}\right);$$

$$E=\frac{H^{\prime }}{\ln S};$$

$$D=\sum_{m=1}^{S}{\left(\frac{N_{m}}{M}\right)}^{2};$$

$$J=\frac{c}{\left(a+b-c\right)}.$$

In the above formulae, *S*_*m*_ = species *m* in a certain community, *a* = the number of chigger species in community A, *b* = the number of chigger species in community B, and *c* = the number of common species in both community A and B. *N*_*m*_ and *M*, the same as before. The value of Jaccard’s similarity index (*J*) ranges from 0 to 1 (*J*: 0–1).

### Analysis of the spatial distribution pattern of dominant chigger species

In combination with the significance test of deviation (*F* test), Iwao’s linear regression model (M* = *α* + *βM*) was used to analyze the spatial distribution pattern of dominant chigger species on *A. squamipes* shrews [10, 15, 16, 24]. In the regression equation M* = *α* + *βM*, when *α* = 0 and *β* = 1 (*F* < *F*_0.05(2, *N*-2)_, *p* > 0.05), the spatial distribution pattern was determined to be the random distribution, and when *α* > 0 and *β* > 1 (*F* > *F*_0.05(2, *N*-2)_, *p* < 0.05), the aggregated distribution [10, 39].

$$M^{*}=\alpha +\beta M(\mathrm{Iwao’}s\;\mathrm{li}\mathrm{near}\;\mathrm{regression});$$

$$F=\frac{\frac{1}{2}\left[N\alpha^{2}+2\alpha (\beta -1)\sum_{i=1}^{N}M_{i}+{\left(\beta -1\right)}^{2}\sum_{i=1}^{N}M_{i}^{2}\right]}{\frac{1}{N-2}\sum_{i=1}^{N}(M_{i}^{*}-\alpha -\beta M_{i}{)}^{2};}$$

$$M_{i}=\frac{1}{N_{i}}\sum_{j=1}^{N_{i}}M_{\mathrm{ij}};$$

$$M_{i}^{*}=M_{i}+\left(\frac{\sigma_{i}^{2}}{M_{i}}-1\right).$$

In the above formulae, *M* and M* represent the mean of chigger individuals and Lloyd’s mean crowding, *α* the intercept and *β* the slope in establishing Iwao’s linear regression. *M*_*i*_, and *M*_i_* stand for the mean and Lloyd’s mean crowding in sampling unit *i*, and *N* is the number of sampling units. *M*_*ij*_ is the chigger individuals on animal host (*A. squamipes*) *j* in sampling unit *i*, *N*_*i*_ the number of animal hosts in sampling unit *i*, and *M*_*i*_, *σ*_*i*_^2^ and *M*_i_* the mean, variance and mean crowding in sampling unit *i*.

### Species abundance distribution of chigger community on *A. squamipes*

The curve tendency of the species abundance distribution of the chigger community on *A. squamipes* was depicted in a semi-logarithmic coordinate system. In the semi-logarithmic coordinate system, the *X*-axis was marked with log intervals based on log_3_N and it represents the chigger individuals in the community, and the *Y*-axis marked with arithmetic scales stands for the number of chigger species in the community [11, 44, 70].

### Species-sample relationship of chigger community on *A. squamipes*

In the chigger community on *A. squamipes* shrews, all the individuals of shrews were randomly divided into several groups with 50 shrews in each group and the remaining 44 shrews in the last group, which represents the sampling units in the analysis of species-sample relationship. The curve of the species-sample relationship of the community was then depicted in a coordinate system in which the *X*-axis was marked with log-transformed individuals of *A. squamipes* shrews and the *Y*-axis was marked with the number of chigger species in the community [39, 70, 72].

### Significance test

A Chi-square test (χ^2^) was used to test the significance of *P*_*m*_, and a nonparametric test was used to test the significance of *MA* and *MI*. All the statistical analyses were performed with version 25.0 of SPSS software.

## Results

### Infestation and community structure of chiggers on *A. squamipes*

**Figure 3:**
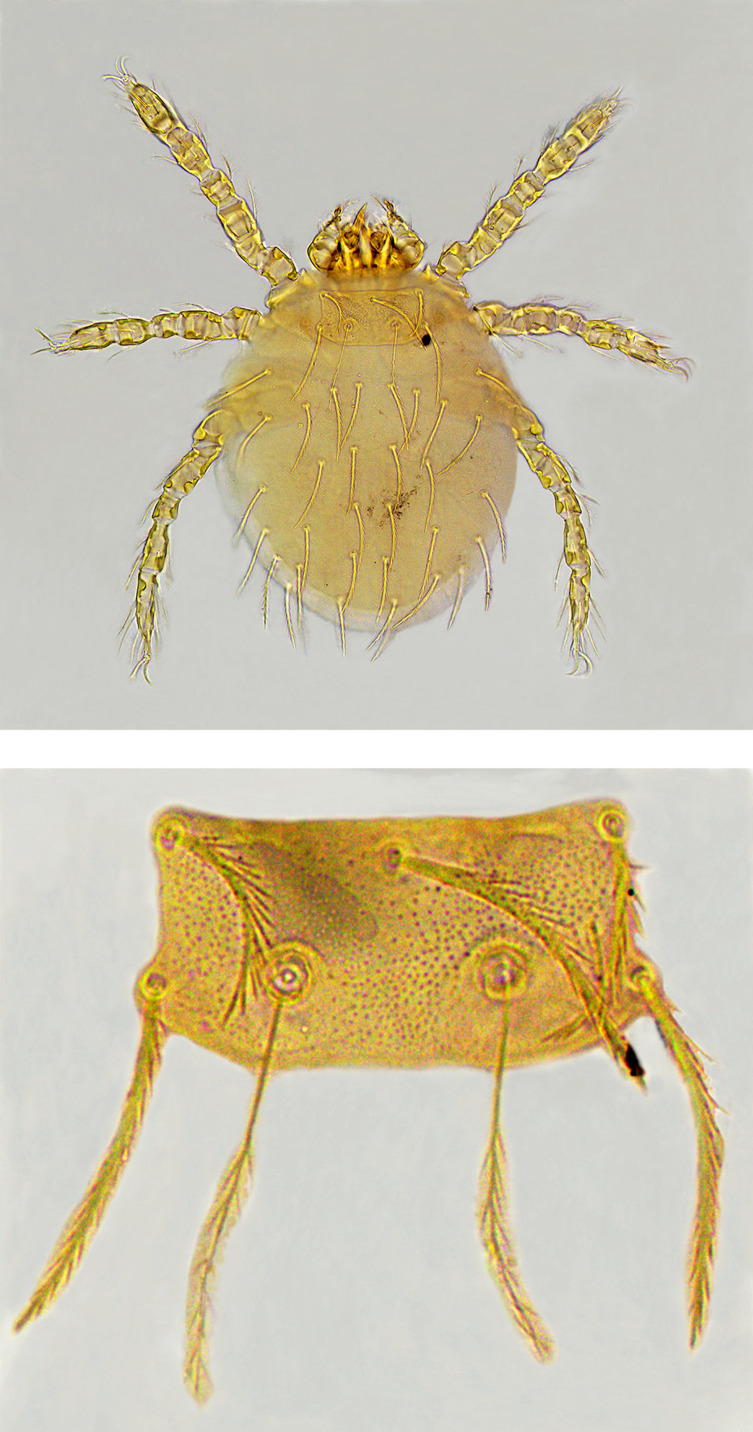
Photos of *Leptotrombidium densipunctatum* (10 × 40), the most dominant chigger species on *Anourosorex squamipes* in Southwest China (top: the whole chigger; bottom: the scutum).

As shown in Table 1 and Figure 1, Chinese mole shrews were captured from 49 of 91 investigation sites (counties), with a total of 3192 chiggers collected from 1694 host shrews. Based on a series of taxonomic structures of chiggers (Table 2), 3169 of 3192 collected chiggers were identified into 72 species and 10 genera in the family Trombiculidae, with high species diversity (Table 3). The remaining 23 chiggers were unidentified because of broken body, covered dirt, unclear structure and suspected new species, and they were not included in the statistical analysis. The overall prevalence (*P*_*m*_), mean abundance (*MA*), and mean intensity (*MI*) of *A. squamipes* with chiggers reached 11.1%, 1.87 and 16.86, respectively. Of 72 chigger species identified, *Leptotrombidium densipunctatum* (Yu *et al.*, 1982) (Fig. 3) was the most dominant with the highest constituent ratio (*C*_*r*_ = 22.1%), and the *C*_*r*_ of every other chigger species was less than 10%. The infestation indices of *L. densipunctatum* on *A. squamipes* were *P*_*m*_ = 4.6%, *MA* = 0.41 and *MI* = 9.10. Based on the 72 identified species and 3169 chigger individuals, the community structure was calculated. The species richness (*S*), Shannon–Wiener diversity index (*H*′), Pielou evenness index (*E*), and Simpson dominance index (*D*) of the chigger community on *A. squamipes* were *S* = 72, *H*′ = 2.90, *E* = 0.68 and *D* = 0.09, respectively.

**Table 3:** Chiggers identified from Chinese mole shrews (*Anourosorex squamipes*) in Southwest China (2001–2019).

Taxonomic taxa of chiggers	Individuals
Family Trombiculidae Ewing, 1929	
Genus *Leptotrombidium* Nagayo *et al.*, 1916	
*L. densipunctatum* Yu *et al.*, 1982	701
*L. rusticum* Yu, Yang & Gong, 1986	284
*L. hiemalis* Yu *et al.*, 1982	220
*L. fujianense* Liao & Wang, 1983	206
*L. longimedium* Wen & Xiang, 1984	191
*L. xiaowei* Wen & Xiang, 1984	167
*L. sinicum* Yu *et al.*, 1981	166
*L. deliense* (Walch, 1922)	144
*L. eothenomydis* Yu & Yang, 1986	120
*L. sialkotense* Vercammen-Grandjean & Langston, 1976	52
*L. shuqui* Wen & Xiang, 1984	38
*L. spicanisetum* Yu *et al.*, 1986	23
*L. bambicola* Wen & Xiang, 1984	25
*L. scutellare* (Nagayo *et al.*, 1921)	20
*L. rubellum* Wang & Liao, 1984	17
*L. longchuanense* Yu & al., 1981	17
*L. kunmingense* Wen & Xiang, 1984	13
*L. lianghense* Yu *et al.*, 1983	10
*L. gongshanense* Yu *et al.*, 1981	8
*L. yongshengense* Yu & Yang, 1986	6
*L. suense* Wen, 1984	6
*L. kitasatoi* (Fukuzumi & Obata, 1956)	6
*L. bishanense* Yu *et al.*, 1986	6
*L. guzhangense* Wang *et al.*, 1985	6
*L. imphalum* Vercammen-Grandjean & Langston, 1975	4
*L. xiaguanense* Yu & Yang, 1981	3
*L. quadrifurcatum* Wen & Xiang, 1984	3
*L. akamushi* (Brumpt, 1910)	3
*L. dognluoense* Wang *et al.*, 1981	3
*L. baoshui* Wen & Xiang, 1984	3
*L. wangi* Yu *et al.*, 1986	2
*L. sheshui* Wen & Xiang, 1984	2
*L. allosetum* Wang *et al.*, 1981	2
*L. kawamurai* (Fukuzumi & Obata, 1953)	2
*L. apodevrieri* Wen & Xiang, 1984	2
*L. ejingshanense* Yu *et al.*, 1982	2
*L. alpinum* Yu & Yang, 1986	2
*L. qujingense* Yu *et al.*, 1981	2
*L. chuanxi* Wen *et al.*, 1984	1
*L. shanghaiense* Wen & Lu, 1984	1
*L. taishanicum* Meng *et al.*, 1983	1
*L. pavlovskyi* (Schulger, 1948)	1
*L. muntiaci* Wen & Xiang, 1984	1
*L. hanseni* Traub & Lakshana,1966	1
Genus *Gahrliepia* Oudemans, 1912	
*G. eurypunctata* Jeu, Yu & Wan, 1983	312
*G. longipedalis* Yu & Yang, 1986	77
*G. tenella* Traub & Morrow, 1955	57
*G. latiscutata* Chen & Fan, 1981	49
*G. radiopunctata* Hsu *et al.*, 1965	46
*G. yangchenensis* Chen et Hsu, 1957	42
*G. deqinensis* Yu & Yang, 1982	19
*G. yunnanensis* Hsu *et al.*, 1965	14
*G. chekiangensis* Chu, 1964	8
*G. silvatica* Yu & Yang, 1982	4
*G. zhongwoi* Wen & Xiang, 1984	3
*G. agrariusia* Hsu *et al.*, 1965	2
*G. lamella* Chen *et al.*, 1980	2
*G. octosetosa* Chen *et al.*, 1956	2
*G. xiaowoi* Wen & Xiang, 1984	1
*G. miyi* Wen & Song, 1984	1
Genus *Trombiculindus* Radford, 1948	
*T. nujiange* Wen & Xiang, 1984	1
*T. cuneatus* Traub & Evans, 1951	1
Genus *Neotrombicula* Hirst, 1925	
*N. longmenis* Wen & Xiang, 1984	4
*N. japonica* (Tanaka *et al.*, 1930)	3
Genus *Ascoschoengastia* Ewing, 1945	
*A. lorius* (Gunther, 1939)	1
Genus *Schoengastiella* Hirst, 1915	
*S. ligula* Radford, 1946	2
Genus *Walchia* Ewing, 1931	
*W. ewingi* (Fuller, 1949)	2
*W. kor* (Chen & Hsu, 1957)	1
Genus *Herpetacarus* Vercammen-Grandjean, 1960	
*H. hastoclavus* Yu *et al.*, 1979	1
*H. aristoclavus* Yu *et al.*, 1979	1
Genus *Huabangsha* Wen *et al.*, 1980	
*H. megachela* Wen *et al.*, 1980	1
Genus *Intermedialia* Yu *et al.*, 1979	
*I. hegu* (Yu, Yang & Wu, 1979)	19

### Fluctuation of chigger infestation in different environments

The chigger infestation and community structure on *A. squamipes* fluctuated in different environments such as latitudes, altitudes, habitats, and landscapes. The species richness of chiggers at latitude 24–26° N was the highest (*S* = 56) with higher infestations (*P*_*m*_ = 60.0%, *MA* = 15.19 and *MI* = 25.31) than at other latitudes (*p* < 0.001) (Table 4). The species similarity of chiggers was low among different latitudes (*J* < 0.5).

**Table 4:** Infestations of *Anourosorex squamipes* shrews with chiggers in different environments in Southwest China (2001–2019).

Different environments		Examined shrews		Species richness and infestation of chiggers on *A. squamipes*				
		Total	Infested	*S*	Number of individuals	*P*_*m*_ (%)	*MA*	*MI*
Latitude (°N)	< 24	27	2	2	2	7.4	0.07	1
	24–26	140	84	56	2126	60.0	15.19	25.31
	26–28	338	38	15	292	11.2	0.86	7.68
	≥ 28	1189	64	16	749	5.4	0.63	11.7
	Total	1694	188	89	3169	11.1	1.87	16.86
Altitude (m)	<1000	1192	64	17	749	5.4	0.63	11.7
	1000–2000	379	69	30	854	18.2	2.25	12.38
	2000–3000	109	55	50	1566	50.5	14.37	28.47
	≥ 3000	14	0	0	0	0	0	0
	Total	1694	188	97	3169	11.1	1.87	16.86
Habitats	Woodlands	133	12	12	69	9.0	0.52	5.75
	Farmlands	547	81	40	800	14.8	1.46	9.88
	Bushes	541	83	54	2250	15.3	4.16	27.11
	Residential areas	473	12	5	50	2.5	0.11	4.17
	Total	1694	188	111	3169	11.1	1.87	16.86
Landscapes	Flatland landscape	1127	56	13	479	5.0	0.42	8.55
	Mountainous landscape	567	132	68	2690	23.3	4.74	20.38
	Total	1694	188	81	3169	11.1	1.87	16.86

From low to high altitudes, the species richness of chiggers on *A. squamipes* showed a parabolic trend, with a peak (*S* = 50) at the middle altitude (2000–3000 m). The infestation indices of the chiggers were also the highest at 2000–3000 m (*P*_*m*_ = 50.5%, *MA* = 14.37 and *MI* = 28.47, *p* < 0.001). No chiggers were found on *A. squamipes* shrews at the high altitudes (≥3000 m) (Table 4). The species similarity of chiggers was low among different altitudes (*J* < 0.5).

The infestation of *A. squamipes* with chiggers also fluctuated in different habitats. The chiggers collected from *A. squamipes* shrews in the bush habitat accounted for 71.0% (2250/3169) of the total identified mites (Table 4). All the species richness and infestation indices of chiggers in the bush habitat (*S* = 54, *P*_*m*_ = 15.3%, *MA* = 4.16 and *MI* = 27.11) were higher than those in other types of habitats, and the species richness and infestation indices were the lowest in residential areas (houses, stables, barns and nearby surroundings) in comparison with the other three types of habitats (bushes, woodlands, and farmlands) (Table 4, *p* < 0.05). The species similarity of chiggers was low among different habitats (*J* < 0.5).

As shown in Table 4, the species richness and infestation indices of chiggers on *A. squamipes* in the mountainous landscape (*S* = 68, *P*_*m*_ = 23.3%, *MA* = 4.74 and *MI* = 20.38) were much higher than those in the flatland landscape (*S* = 13, *P*_*m*_ = 5.0%, *MA* = 0.42 and *MI* = 8.55, *p* < 0.001). The species similarity of chiggers was very low between two landscapes (*J* = 0.10).

### Fluctuation of chigger infestation on different sexes and ages of hosts

The prevalence (*P*_*m*_ = 13.5%) and mean abundance (*MA* = 2.28) of chiggers on female *A. squamipes* shrews (hosts) were higher than those on male hosts (*P*_*m*_ = 8.2%, *MA* = 1.39) (*p* < 0.05). The mean intensity (*MI*) on male hosts was higher than that on females, but the difference was not statistically significant (*p* > 0.05). The species of chiggers on female shrews (60 species) were higher than those on male shrews (41 species) (Table 5). The chigger species on different sexes of *A. squamipes* showed low similarity (*J* = 0.38).

**Table 5:** Chigger infestation on different sexes and ages of *Anourosorex squamipes* shrews in Southwest China (2001–2019).

Sexes and ages of shrews		Examined shrews		Species richness and infestation of chiggers on *A. squamipes*				
		Total	Infested	*S*	Number of individuals	*P*_*m*_ (%)	*MA*	*MI*
Sexes	Female	981	132	60	2232	13.5	2.28	16.91
	Male	671	55	41	936	8.2	1.39	17.02
	Total	1652	187	101	3168	11.3	1.92	16.94
Ages	Juvenile	46	11	12	70	23.9	1.52	6.36
	Adult	1620	171	69	2855	10.6	1.76	16.70
	Total	1666	182	81	2925	10.9	1.76	16.07

Note: Animal hosts without records of sex and age were not included in this table.

The prevalence of chiggers on adult *A. squamipes* hosts (*P*_*m*_ = 10.6%) was lower than that on juvenile hosts (*P*_*m*_ = 23.9%), but the mean abundance and mean intensity on adult hosts (*MA* = 1.76) were higher than those on juvenile hosts (*MA* = 1.52) (*p* < 0.05). The mean intensity (*MI*) on adult hosts was higher than that on juvenile hosts, but the difference was of not statistically significant (*p* > 0.05). Adult shrews harbored more species (69 species) than juvenile shrews (12 species) (Table 5). The chigger species on different ages of *A. squamipes* showed very low similarity (*J* = 0.17).

### Spatial distribution pattern of dominant chigger species on *A. squamipes*

Of 72 chigger species, *L. densipunctatum* was the most dominant (Fig. 3). In combination of the significance test of deviation (*F* test), Iwao’s regression analysis was used to analyze the spatial distribution pattern of *L. densipunctatum* on *A. squamipes* (Table 6). Based on the calculation of mean (*M*_*i*_), variance (*σ*^2^) and Lloyd’s mean crowding (*M*_i_*) in each sampling unit, the linear regression equation was established as M* = 0.173 + 1.054 M (*r* = 0.98, *p* < 0.001), where both *α* and *β* (*α* = 0.173, *β* = 1.054) exceeded the boundary values (0 and 1) for the determination of aggregated distribution with *F* > *F*_0.05 (2, 20)_ and *p* < 0.05 (*F* = 4.61, *F*_0.05 (2, 20)_ = 3.49) in *F* test.

**Table 6:** The calculated mean (*M*_*i*_), variance (*σ*^*2*^) and Lloyd’s mean crowding (*M*_i_*) in different sampling units in Iwao’s linear regression analysis and the significance test of deviation (*F* test).

Sites	*M*_*i*_	*σ* ^*2*^	*M*_*i*_ *	*M*_*i*_ * **-** *α* **-** *βM*_*i*_
1	5.79	7.93	6.16	−0.10
2	2.08	3.05	2.55	0.18
3	1.18	1.88	1.7	0.35
4	0	0	0	−0.17
5	0.14	0.27	1.07	0.75
6	0	0	0	−0.17
7	0	0	0	−0.17
8	4.35	5.84	4.70	−0.07
9	0	0	0	−0.17
10	0	0	0	−0.17
11	0.07	0.13	0.93	0.68
12	0	0	0	−0.17
13	0.01	0.01	0.10	0.82
14	0	0	0	−0.17
15	0	0	0	−0.17
16	0	0	0	−0.17
17	0	0	0	−0.17
18	0	0	0	−0.17
19	0	0	0	−0.17
20	0	0	0	−0.17
21	0	0	0	−0.17
22	0	0	0	−0.17

Note: Each sample unit represents the following counties: 1 = LH + RL; 2 = CY + GM + YD; 3 = DL; 4 = JP + MG; 5 = GS + LP + YL + WX; 6 = FY; 7 = QJ + SJ; 8 = HX + HS + DYu + LLi; 9 = XX + ZS; 10 = JS; 11 = MN + YY + MLi; 12 = XZ + PS; 13 = QW + SZh + HY; 14 = JY + LX; 15 = WY + ZZ + RS; 16 = JT; 17 = FC; 18 = PC + XH; 19 = DY + AY + WuS + TN; 20 = JJ; 21 = ZX + DJ + CS; 22 = YuY + WZ.

### Species abundance distribution of chiggers on *A. squamipes*

**Figure 4:**
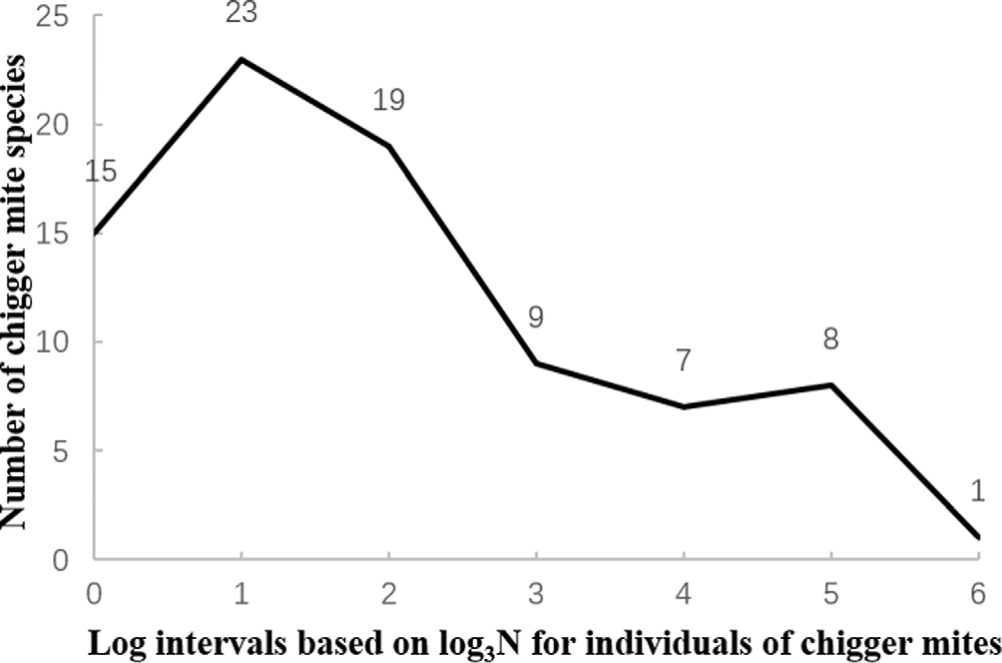
The species abundance distribution of the chigger community on *Anourosorex squamipes* between chigger individuals and their corresponding species in Southwest China (2001–2019).

In a semi-logarithmic coordinate system, the species abundance distribution of the chigger community on *A. squamipes* was depicted (Table 7). The curve of the species abundance distribution showed a gradually descending tendency from the rare chigger species (the highest point at *Y*-axis) to the dominant species (Fig. 4). The majority of chigger species (23 species) at the highest point of the *Y*-axis were rare species and a few mite species with abundant individuals (e.g., *L. densipunctatum*) were dominant (Table 7, Fig. 4).

**Table 7:** Species abundance distribution of the chigger community on *Anourosorex squamipes* in Southwest China (2001–2019).

Log intervals	Individual ranges in each log interval	Midpoint values of each individual range	Actual chigger species
0	0–1	1	15
1	2–4	3	23
2	5–13	9	9
3	14–40	27	9
4	41–121	81	7
5	122–364	243	8
6	365–1093	729	1

### Species-sample relationship of chiggers on *A. squamipes*

**Figure 5:**
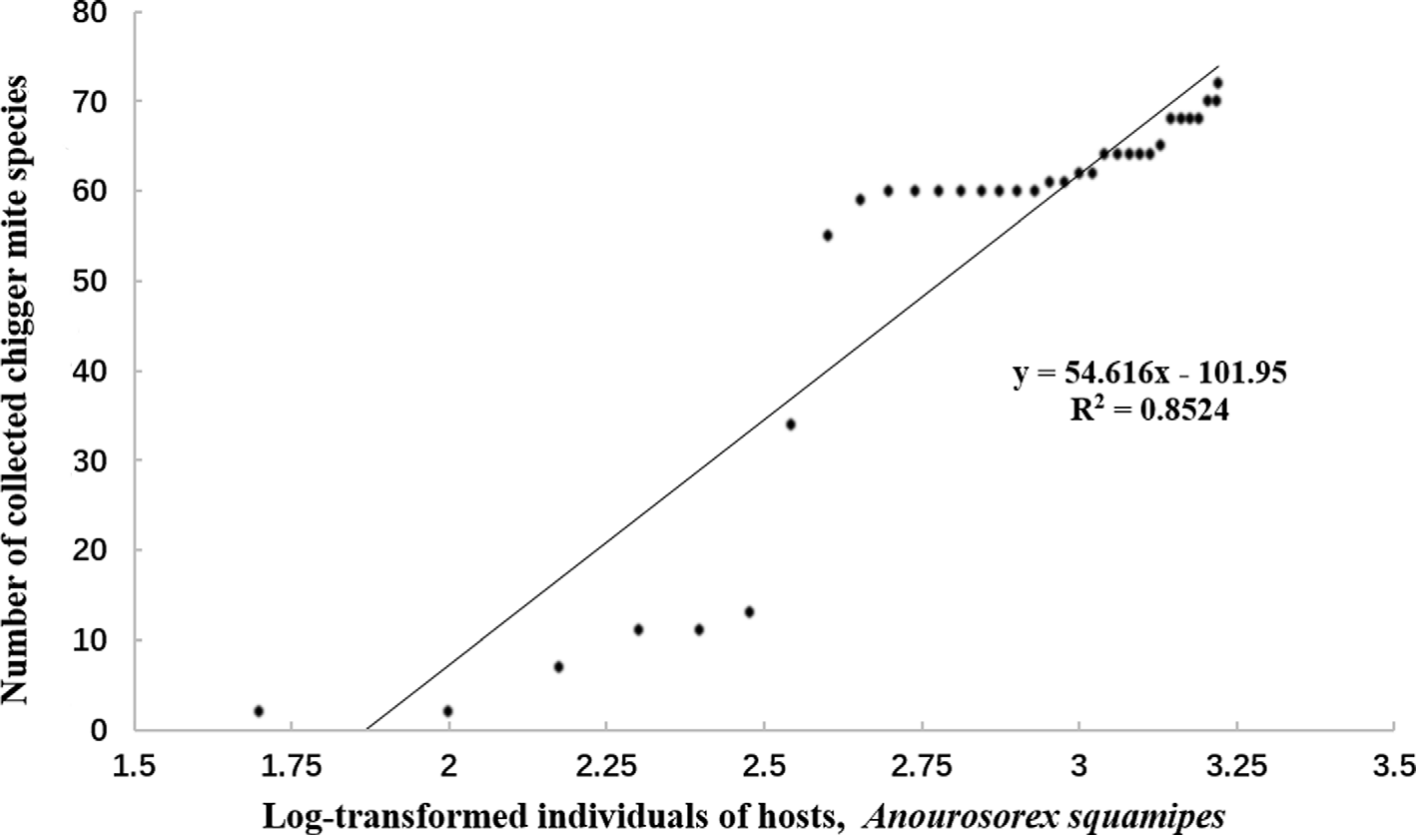
The species-sample relationship of chiggers on *Anourosorex squamipes* shrews between the log-transformed host individuals (shrews) and the number of the chigger species in Southwest China (2001–2019).

The curve of the species-sample relationship of chiggers on *A. squamipes* shrews showed that the number of chigger species at the *Y*-axis increased with the increase of host individuals (shrews) at the *X*-axis. When the logarithm-transformed host individuals (host samples) were at 3.23 scale of the *X*-axis, which corresponds to all the hosts collected (1694 *A. squamipes* shrews), the number of chigger species at the *Y*-axis was still increasing with a continuous “going-up” tendency on the curve of species-sample relationship (Fig. 5). A positive linear correlation existed between the logarithm-transformed host individuals and the number of chigger species (*r* = 0.92).

## Discussion

### Species diversity and infestation of chiggers on *A. squamipes*

In the present study, 72 chigger species with 3169 individuals were collected from 1694 *A. squamipes* shrews in Southwest China. The 72 chigger species identified from such a single species of insectivore in Southwest China even exceeded the total mite species identified from various species of host animals in some provinces of China. For example, a total of 24 chigger species recorded in Shandong Province, 53 species in Fujian Province and 47 species in Hubei Province [7, 55, 64]. These findings indicate that *A. squamipes* has a high potential to harbor many chigger species with high species diversity. Southwest China covers five provincial regions of China and it is a very wide geographical region with different altitudes and landscapes, complex topography and different climate types [1]. Many studies have proven that the host specificity of chiggers is quite low. The same chigger species can infest different species of animal hosts and different chigger species can infest the same host species because of the low host specificity and frequent cross-infestation of chiggers [41, 68]. The species composition of chiggers on the same host species often fluctuates in different geographical regions and ecological environments because of low host specificity [5, 40]. High species diversity of chiggers on *A. squamipes* may be associated with the biological characteristics of *A. squamipes*, the low host specificity and cross-infestation of chiggers, the wide geographical region of Southwest China and its complex topography with different climate types [5, 43, 65]. In addition, the high species diversity of chiggers on *A. squamipes* may also be related to the large number of host samples (1694 *A. squamipes* shrews) collected from different areas of Southwest China, which increases the chances of collecting certain rare mite species [28, 43].

Although *A. squamipes* is an insectivore species, it often co-exists in the same geographic region, landscape and habitat with many rodent species such as *Rattus norvegicus*, *R. tanezumi* and *Eothenomys miletus* [41, 42]. A previous study showed that a total of 61 chigger species were identified from the brown rat (Norway rat, *Rattus norvegicus*) in Yunnan Province, Southwest China and the infestation indices of chiggers on *R. norvegicus* (*P*_*m*_ = 13.4%, *MA* = 1.27 and *MI* = 9.49) are close to those on *A. squamipes* (*P*_*m*_ = 11.1%, *MA* = 1.87 and *MI* = 16.86) in the present study [5]. In contrast, a total of 131 species of chigger mites were previously identified from the oriental house rat (Asian house rat, *R. tanezumi*) in Yunnan, with higher infestation indices (*P*_*m*_ = 20.9%, *MA* = 6.20 and *MI* = 29.80) [6], and 175 chigger species were identified from the large Chinese vole (large oriental vole or Yunnan red-backed vole, *E. miletus*) in Southwest China, with much higher infestation (*MA* = 20.24, 49850/2463) [43]. The species diversity (*S* = 71) and infestation of chiggers on *A. squamipes* are obviously lower than those on *R. tanezumi* and *E. miletus* [6, 43]. The results indicate that different species of small mammals (rodents and shrews) have different potential to harbor chiggers with different species diversity and infestation of the mites, which is associated with the different biological characteristics of different host species [5, 6, 40].

### Infestation of *A. squamipes* with chiggers in different environments

The results of the present study showed that the infestation of *A. squamipes* with chiggers fluctuated in different environments (latitudes, altitudes, habitats, and landscapes). The species richness of chiggers was the highest at latitude 24–26° N with higher infestation than at other latitudes. The mite species richness showed a parabolic trend with the highest species richness and infestation at the middle altitude. In comparison with other three types of habitats (bushes, woodlands, and farmlands), the species richness and infestation indices of chiggers on *A. squamipes* were the lowest in residential areas (houses, stables, barns, and nearby surroundings) (Table 4). The species richness and infestation indices in the mountainous landscape were higher than those in the flatland landscape. The species similarities of chigger were low in different latitudes, altitudes, habitats, and landscapes (*J* < 0.5). The results indicate that the infestation of chiggers on the same host species (*A. squamipes*) was not stable and fluctuates in different environments, which may be related to the environmental heterogeneity and the low host specificity of chiggers [6, 40, 69]. Different chigger species usually have different adaptability to different environmental conditions [30, 35, 68]. There are usually different biodiversity, vegetation, and climate factors (temperature, humidity and sunshine, etc.) in different latitudes, altitudes, habitats, and landscapes, and this may be suitable to the growth, development, and reproduction of different chigger species [63, 65]. For example, there are usually far more species of plants and animals with much higher biodiversity and vegetation in outdoor habitats such as bushes, woodlands, and farmlands than in indoor and residential habitats such as houses, stables, barns, and nearby surroundings [6, 40, 62], and this may explain why the species richness and infestation indices of chiggers on *A. squamipes* in the habitats of bushes, woodlands and farmlands are higher than those in the residential areas (Table 4). The infestation fluctuation of chiggers in different environments also reflects the influence of environmental factors on chiggers [30, 35, 63]. Besides the influence of environmental heterogeneity, the low host specificity of chiggers may also contribute to the fluctuation of chigger infestation in different environments. As a group of ectoparasites, chiggers usually have a wide range of hosts with low host specificity. Most chigger species have not established stable and fixed parasitism with their hosts, and therefore mite infestations on the same host species would greatly fluctuate in different environments with very low species similarities [5, 42, 48]. A certain chigger species can parasitize different hosts and a certain host species can harbor different chigger species as well. Therefore, the same host species (e.g., *A. squamipes*) may harbor different chigger species with different mite burdens under different environmental conditions [6, 30, 42].

### Chigger infestation on different sexes and ages of hosts

The results of the present study showed that the prevalence and mean abundance of chiggers on female *A. squamipes* shrews (hosts) were higher than those on male hosts. The number of chigger species on female shrews was also higher than those on male shrews (Table 5). The chigger prevalence on adult shrews was lower than that on juvenile shrews, but the mean abundance and mean intensity on adult shrews were higher than those on juvenile shrews. Adult shrews harbored more species than juvenile shrews (Table 5). The species similarities of chiggers were low on different sexes and ages of hosts with *J* = 0.38 and *J* = 0.17 which are much lower than 0.5, the half value of species similarity. The results indicate that chigger infestation is quite different on different sexes and ages of hosts, and this reflects the sex-bias and age-bias of the shrews when infested with chiggers. Previously there have been some reports on the sex-bias and age-bias of rodents and some other small mammals when infested with ectoparasites (including chigger mites), but their results were inconsistent. Studies have shown that male and adult hosts are more susceptible to ectoparasites with more parasite species and heavier infestation, but other reports found the opposite result [6, 28, 62]. Despite this, sex-bias and age-bias do exist in some small mammals when infested with ectoparasites. The different biological characteristics of different sexes and ages of animal hosts are often considered to be the main reasons underlying sex-bias and age-bias [20, 23].

### Dominant chigger species and their spatial distribution pattern

Spatial distribution patterns are important in the study of animal and plant ecology, and usually include three pattern types: uniform, random and aggregated distributions [43, 52]. There are a series of methods to determine the spatial distribution pattern of a certain population, and Iwao’s regression method in combination with the significance test of deviation (*F* test) is one of them [10, 39]. The present study used Iwao’s regression with the significance test of deviation to analyze the spatial distribution pattern of *L. densipunctatum* which is the most dominant chigger species on *A. squamipes*. The result showed that both *α* and *β* values (*α* = 0.173, *β* = 1.054) exceeded the boundary values (*F* > *F*_0.05 (2, 20)_, *p* < 0.05) in *F* test, and therefore the spatial distribution of *L. densipunctatum* is considered the aggregated distribution among different individuals of its hosts, *A. squamipes* shrews. The aggregated pattern is common in parasites and the findings in our study are highly consistent with various previous reports [3, 13, 59]. The aggregated pattern suggests that chigger distribution among different individuals of the shrew hosts is quite uneven. Some shrews harbor many individuals on their body surface forming clumps of mites, and other shrews have few or no mites. Aggregated distribution is beneficial to survival, mating, and reproduction of parasites [13, 21, 46].

### Species abundance distribution of chiggers on *A. squamipes*

Species abundance distribution is an important notion in community ecology as it illustrates the relationship between the number of species and individuals in a community [11, 33, 72]. Our results showed that the species abundance distribution of chiggers on *A. squamipes* conformed to lognormal distribution, indicating that most chigger species are rare mite species, and few chigger species are the dominant mite species with abundant individuals. This result is highly consistent with findings reported in previous studies [6, 39].

### Species-sample relationship of chiggers on *A. squamipes*

The species-sample relationship is used to illustrate the relationship between the sample and number of species in a certain community [70, 72]. In the present study, the *X*-axis was marked with the log-transformed individuals of *A. squamipes* shrews (host samples) and the *Y*-axis with the number of chigger species. Theoretically the number of species at the Y-axis would quickly increase with the increase of host samples at the beginning. When the increase of host samples goes on, the increase of species would gradually slow down, and ultimately get close to stopping and form a stable “platform” stage when the host samples become big enough [32, 43, 72]. In the present study, however, the number of chigger species at the *Y*-axis still kept increasing without the appearance of a stable “platform” stage when the host samples became quite big, 1694 *A. squamipes* shrews (Fig. 5). The continuous increasing tendency of the species-sample curve implies that 1,694 *A. squamipes* shrews (host samples) in the present study are still unable to reflect the complete species composition of chiggers on *A. squamipes* in the whole of Southwest China. If host samples infinitely keep increasing, more and more chigger species would be found. As mentioned above, the species composition and infestation of *A. squamipes* with chiggers vary in different environments with obvious heterogeneity. To reflect the complete species composition of chiggers in a very large geographical region like Southwest China, a large host sample is recommended [19, 28].

## Conflict of interest

The authors declare that they have no conflict of interest.
